# Increased GDF9 and BMP15 mRNA levels in cumulus granulosa cells correlate with oocyte maturation, fertilization, and embryo quality in humans

**DOI:** 10.1186/1477-7827-12-81

**Published:** 2014-08-20

**Authors:** Yi Li, Rui-Qi Li, Song-Bang Ou, Ning-Feng Zhang, Ling Ren, Li-Na Wei, Qing-Xue Zhang, Dong-Zi Yang

**Affiliations:** Reproductive Medicine Research Center, Memorial Hospital of Sun Yat-Sen University, 107 Yan-jiang-xi Road, Guangzhou, 510120 China; Ultrasound Diagnostic Center, First Affiliated Hospital of Gannan Medical Collage, 23 Qing-nian Road, Gangzhou, 341000 China; Reproductive Medicine Research Center, Sixth Affiliated Hospital of Sun Yat-Sen University, 17 Shou-gou-ling Road, Guangzhou, 510655 China

**Keywords:** Growth differentiation factor 9, Bone morphogenetic protein 15, *In vitro* fertilization and embryo transfer

## Abstract

**Background:**

Oocyte secreted factors (OSFs), including growth differentiation factor 9 (GDF9) and bone morphogenetic protein 15 (BMP15), play an important role in the process of follicular development and oocyte maturation. Since OSFs are expressed in oocytes and cumulus granulosa cells, the aim of the present study was to explore whether the expression levels of *GDF9* and *BMP15* mRNAs in cumulus granulosa cells can be used as molecular markers for predicting oocyte developmental potential.

**Methods:**

Cumulus cells of 2426 cumulus-oocyte complexes were collected from 196 female patients who underwent intracytoplasmic sperm injection (ICSI) and were used for mRNA detection on the egg retrieval day. Pearson correlation analysis was used to analyze the correlation between OSF expression and general physiological parameters. Partial correlation analysis was used to analyze the correlation between OSF expression and oocyte developmental potential. Covariance analysis was used to compare OSF expression among different groups. Receiver operating characteristic curves were used to examine the diagnostic value of *GDF9* and *BMP15* mRNA for predicting pregnancy.

**Results:**

The expression levels of *GDF9* and *BMP15* mRNAs were significantly associated with age, body mass index (BMI), oocyte maturation, normal fertilization, and cleavage rate (*P* < 0.05). The expression levels of *GDF9* and *BMP15* mRNAs in the group with high-quality embryos were significantly higher than those in the group without high-quality embryos (*P* < 0.05). The expression levels of *GDF9* and *BMP15* mRNAs in the pregnancy group were significantly higher than those in the nonpregnancy group (*P* < 0.05). The cut-off value of *GDF9* mRNA for predicting pregnancy was 4.82, with a sensitivity of 82% and a specificity of 64%. The cut-off value of *BMP15* mRNA for predicting pregnancy was 2.60, with a sensitivity of 78% and a specificity of 52%.

**Conclusions:**

The expression levels of *GDF9* and *BMP15* mRNAs were closely associated with oocyte maturation, fertilization, embryo quality, and pregnancy outcome; therefore, *GDF9* and *BMP15* mRNAs in cumulus granulosa cells may be considered as new molecular markers for predicting oocyte developmental potential.

## Background

The oocyte developmental potential is one of the key factors for determining the successful rate of *in vitro* fertilization and embryo transfer (IVF-ET). The accurate evaluation of oocyte developmental potential is an important issue in assisted reproduction. The traditional method uses morphological scoring. The advantages of morphological scoring lie in its simplicity, convenience, and fast speed [1–3]. However, the main shortcoming of this method is that it depends too much on the abilities of the technician, so it is difficult to reach a uniform standard. In some cases, the morphological scoring may not accurately reflect the oocyte developmental potential and embryo quality [4].

Recently, global assessment strategies including genomic, transcriptomic, and proteomic approaches have been applied in assisted reproduction [5]. These strategies aim to present a “molecular profile” of embryo development by detecting the chemical components in the oocyte, granulosa cells, follicular fluid, and embryo culture medium. These methods pave a new way to enhance the accuracy of the oocyte developmental potential.

Granulosa cells are distributed on the follicular wall (mural granulosa cells) or closely adjacent to the oocyte (cumulus granulosa cells). The physiological function of mural granulosa cells is predominantly related to hormone secretion. Cumulus granulosa cells often exchange biological signals with oocytes through the gap junction [6–8]. There is a mutual communication between cumulus granulosa cells and the oocyte. Recent studies have shown that the expression levels of some genes in cumulus granulosa cells are helpful for predicting the oocyte developmental potential, such as hyaluronic acid synthase 2 (*HAS2*), gremlin 1 (*GREM1*), etc. [9, 10]. Since cumulus granulosa cells are by-products of intracytoplasmic sperm injection (ICSI) and the method of detecting gene expression in cumulus granulosa cells does not affect the oocyte developmental potential, it is very possible to screen suitable potential biomarkers for predicting oocyte developmental potential in IVF clinics.

Oocyte secreted factors (OSFs) include growth differentiation factor 9 (GDF9) and bone morphogenetic protein 15 (BMP15). Both factors play an important role in the process of follicular development from the recruitment of the primordial follicle to ovulation and even in corpus luteum formation [11–13]. OSFs contribute to promoting the proliferation and differentiation of granulosa cells and oocyte maturation through paracrine and autocrine signaling pathways. Previous studies have indicated that higher GDF9 and BMP15 levels in the follicular fluid are significantly associated with oocyte maturation and embryo quality [14–16]. *In vitro* studies have shown that GDF9 and BMP15 may stimulate M-phase-promoting factor (MPF) and mitogen-activated protein kinase (MAPK) activities in oocytes and improve oocyte quality and subsequent developmental potential [17, 18]. In addition, it has been confirmed by many studies that OSFs are expressed both in oocytes and cumulus granulosa cells [19–21]. The aims of the present study were to detect the expression levels of *GDF9* and *BMP15* mRNAs in cumulus granulosa cells and to analyze the correlation between their expression levels and the oocyte developmental potential.

## Methods

### Study design

This study was approved by the Institutional Review Board of Sun Yat-Sen University in March 2012 (NO. E2012003). All the subjects signed the informed consent. This retrospective study was conducted at the Center for Reproductive Medicine in Memorial Hospital of Sun Yat-Sen University from September 2012 to April 2013. In total, 196 women who underwent ICSI because their husbands were diagnosed as having severe oligospermia and asthenospermia (total number <1 × 10^6^/ml, motility <5%) were recruited into this study. The general information of the patients is presented in Table 1. The inclusion criteria for all patients included a long protocol for ovarian stimulation, age  ≤45 years, body mass index (BMI) of 17–35 kg/m^2^, and basal follicle-stimulating hormone (FSH) level ≤IU/L. The exclusion criteria included a history of previous poor response, recurrent implantation failure (failed to achieve a pregnancy after three or more cycles), submucosal fibroids, intrauterine adhesion, congenital uterine malformation, hydrosalpinx, ovarian endometriomas >3 cm in diameter, and polycystic ovarian syndrome.

**Table 1:** **General information for all the subjects**

Variable	Value
*Cases*	196
*Number of COCs*	2426
*Age (years)*	32.81 ± 5.16
*BMI (kg/m* ^*2*^ *)*	21.43 ± 3.20
*Antral follicle count (both ovaries)*	13.66 ± 6.82
*Ovary volume (mL)*	5.67 ± 3.02
*FSH (IU/L)*	8.86 ± 4.34
*LH (IU/L)*	5.18 ± 3.26
*E*_*2*_ *(pg/mL)*	56.29 ± 7.89
*T (ng/L)*	1.73 ± 0.92
*PRL (pg/mL)*	16.61 ± 10.01
*Days of stimulation*	10.83 ± 3.42
*Gonadotropin dose (IU)*	2157.83 ± 999.63
*Retrieved oocytes*	12.25 ± 7.93
*Oocyte maturation rate (%)*	83.76 ± 17.54
*Normal fertilization rate (%)*	70.49 ± 24.47
*Cleavage rate (%)*	86.67 ± 21.98
*Pregnancy rate (%)*	51.02

### Ovarian stimulation protocol

All subjects underwent a long protocol for ovarian stimulation. The blood was taken for determining the basal levels of endocrine hormones during the menstrual cycle. Endocrine hormones such as FSH, luteinizing hormone (LH), estradiol (E_2_), testosterone (T), and prolactin (PRL) were measured by an Axsym chemiluminescence detection system (Axsym; Abbott Laboratories, Rungis, France). A gonadotropin-releasing hormone agonist (1.25 mg, Ipsen Pharma Biotech, Paris, France) was subcutaneously injected for pituitary down regulation. Gonadotropin (Gonal-f, 150–300 IU; Merck Serono, Darmstadt, Germany) was subcutaneously injected for ovarian hyperstimulation 2 weeks after downregulation. Human chorionic gonadotropin (hCG, 10,000 IU; Northern Pharma Inc., Rostov-Na-Dony, Russia) was intramuscularly injected when the diameter of at least three dominant follicles was larger than 18 mm. The collection of cumulus-oocyte complexes (COCs) was conducted with a 17G needle under ultrasound monitoring 36 h after hCG injection.

### Quantitative polymerase chain reaction (qPCR)

In total, 2426 COCs retrieved from pooled follicles of individual patients were cultured *in vitro* for 2 h; then, the cumulus granulosa cells were stripped off with visualization under an inverted micoscope after hyaluronidase digestion. The stripped cumulus granulosa cells (1 × 10^2^–5 × 10^3^) were rinsed with phosphate-buffered saline two times and mixed with Trizol (Invitrogen, Grand Island, NY, USA). Total RNA was extracted with Trizol and reversely transcribed into cDNA with the Superscript III kit (Invitrogen, Grand Island, NY, USA). The qPCR was performed with Taqman fluorescent probes and an ABI Prism7700 detection system. The primers for GDF9 (NM_005448), BMP15 (NM_005260), and GAPDH (glyceraldehyde-3-phosphate dehydrogenase) were as follows: GDF9: forward 5′-GGCAAGGCCTCACAGAGGTA-3′, reverse 5′-CGGTAAACCACAGTGGCTCTAAC-3′; BMP15: forward 5′-CTGCTTTGCCTGGCTGTGT-3′, reverse 5′-CAAGGCATAGCCCCAGATTC-3′; GAPDH: forward 5′-CCTGCACCACCAACTGCTTAG-3′, reverse 5′-CAGTCTTCTGGGTGGCAGTGA-3′. GAPDH was used as the endogenous control for normalization. All the PCR conditions consisted of 93°C for 3 min, followed by 40 cycles of 93°C for 45 s and 55°C for 1 min. The 2^-Î”Î”Ct^ algorithm was used to calculate the GDF9 and BMP15 mRNA levels relative to the GAPDH level.

### Oocyte and embryo assessment

Oocyte maturation was examined under an inverted micoscope. If the first polar body was observed in the oocyte cytoplasm, the oocyte was regarded as being at the metaphase II (MII) stage. The oocyte maturation rate refers to the number of MII oocytes divided by the total number of all retrieved oocytes. The oocyte at the MII stage was fertilized with the help of the ICSI procedure. Oocyte fertilization was observed 18–19 h after ICSI. Normal fertilization was confirmed when two pronuclei (2PN) were found in the cytoplasm. The normal fertilization rate refers to the number of fertilized oocytes divided by the total number of all retrieved oocytes. Embryo cleavage was examined 43–45 h after ICSI. Normal embryo cleavage was defined when the fertilized egg developed into an embryo with 4–6 blastomeres. The cleavage rate refers to the number of cleaved zygotes divided by the total number of all zygotes. Embryo evaluation was conducted 67–69 h after oocyte fertilization. A high-quality embryo should consist of 7–9 blastomeres with a uniform size, and the fragment proportion should be less than 10%. If a patient had at least one embryo with the above criteria, she would be included in the group with high-quality embryos. If a patient did not have any embryos with the above criteira, she would be included in the group without high-quality embryos. No more than three embryos were transferred into the uterine cavity on day 3 of *in vitro* culture. Clinical pregnancy was diagnosed when the gestational sac and fetal heart beat were observed under ultrasound 5 weeks after embryo transfer.

### Statistical analysis

The one-sample Kolmogorov-Smirnov test was used to examine the normal distribution of all data. The relative expression levels of *GDF9* and *BMP15* mRNAs were logarithmically transformed into a normal distribution. The data in the tables were expressed as means ± standard deviation (SD). Pearson correlation analysis was used to analyze the correlation between OSF expression and general physiological paratmeters. Partial correlation analysis was used to analyze the correlation between *GDF9* and *BMP15* expression and oocyte developmental potential after age adjustment. Covariance analysis was used to compare *GDF9* and *BMP15* expression in different groups after adjusting for the number of retrieved oocytes. Receiver operating characteristic (ROC) curves were used to examine the diagnostic value of *GDF9* and *BMP15* mRNA for predicting pregnancy. The area under the curve (AUC) represents the probability of correctly identifying the pregnant and nonpregnant cases. Data analysis was conducted with *SPSS* 11.5, and *P* < 0.05 was considered as statistically significant.

## Results

### Correlation between the expression of *GDF9*and *BMP15*mRNAs and physiological parameters

**Figure 1:**
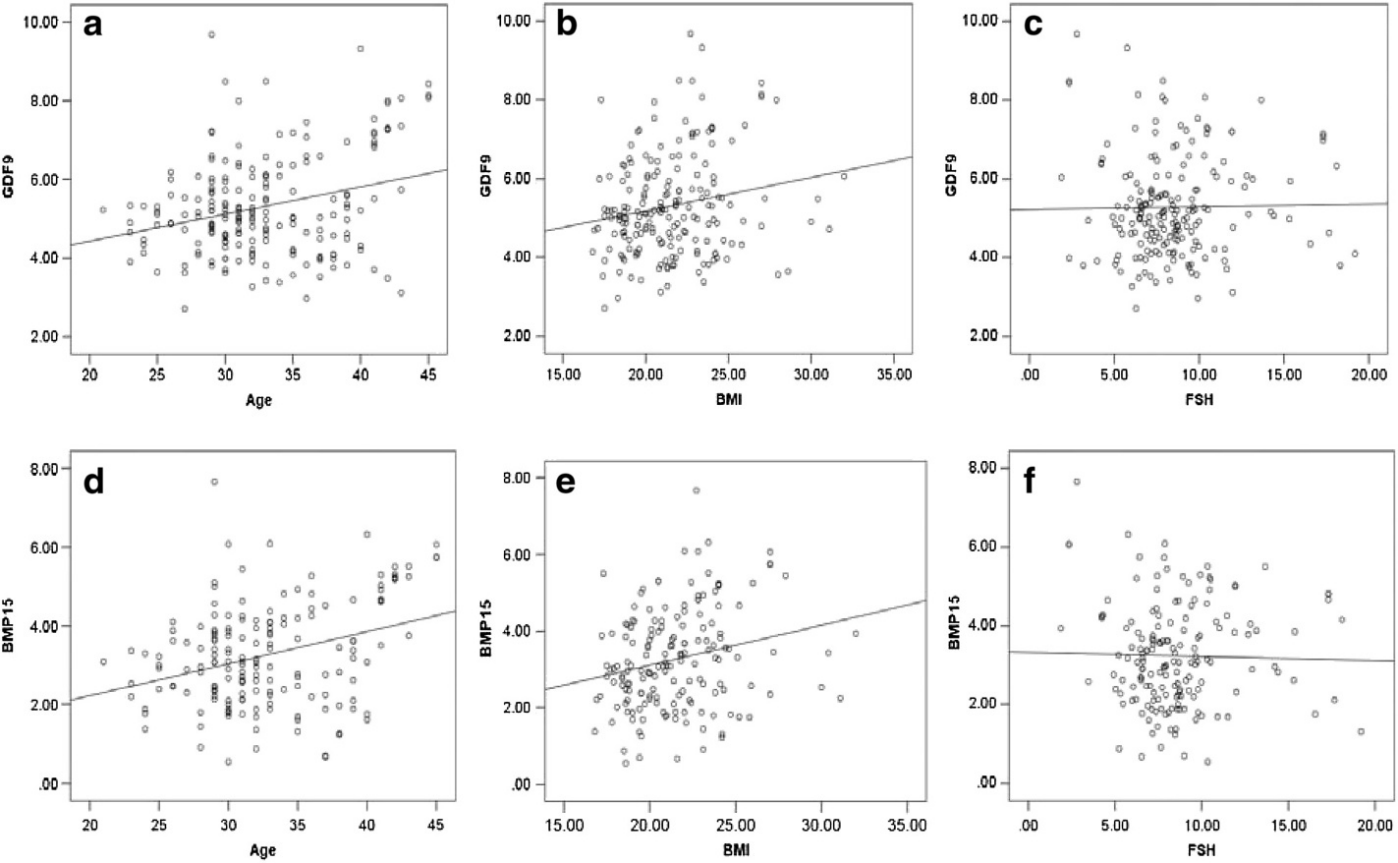
**Pearson analysis was used to analyze the correlation between** ***GDF9*** **and** ***BMP15*** **expression and general parameters in 196 patients who underwent ICSI.** The numbers on the horizzontal-axes refer to the age, BMI, and FSH. The numbers on the y longitudinal axes refer to the relative mRNA expression of GDF9 or BMP15. **(a)** The correlation between age and the relative expression of *GDF9* mRNA. **(b)** The correlation between BMI and the relative expression of *GDF9* mRNA. **(c)** The correlation between FSH and the relative expression of *GDF9* mRNA. **(d)** The correlation between age and the relative expression of *BMP15* mRNA. **(e)** The correlation between BMI and the relative expression of *BMP15* mRNA. **(f)** The correlation between FSH and the relative expression of *BMP15* mRNA.

The expression level of *GDF9* mRNA was significantly associated with age and BMI but not FSH, and the coefficients of correlation were 0.278 (*P* < 0.05), 0.188 (*P* < 0.05), and 0.017 (*P* > 0.05), respectively (Figure 1a–c). Similarly, the expression level of *BMP15* mRNA was also associated with age and BMI but not FSH, and the coefficients of correlation were 0.324 (*P* < 0.05), 0.226 (*P* < 0.05), and 0.024 (*P* > 0.05), respectively (Figure 1d–f).

### Correlation between the expression of *GDF9*and *BMP15*mRNAs and oocyte developmental potential

**Figure 2:**
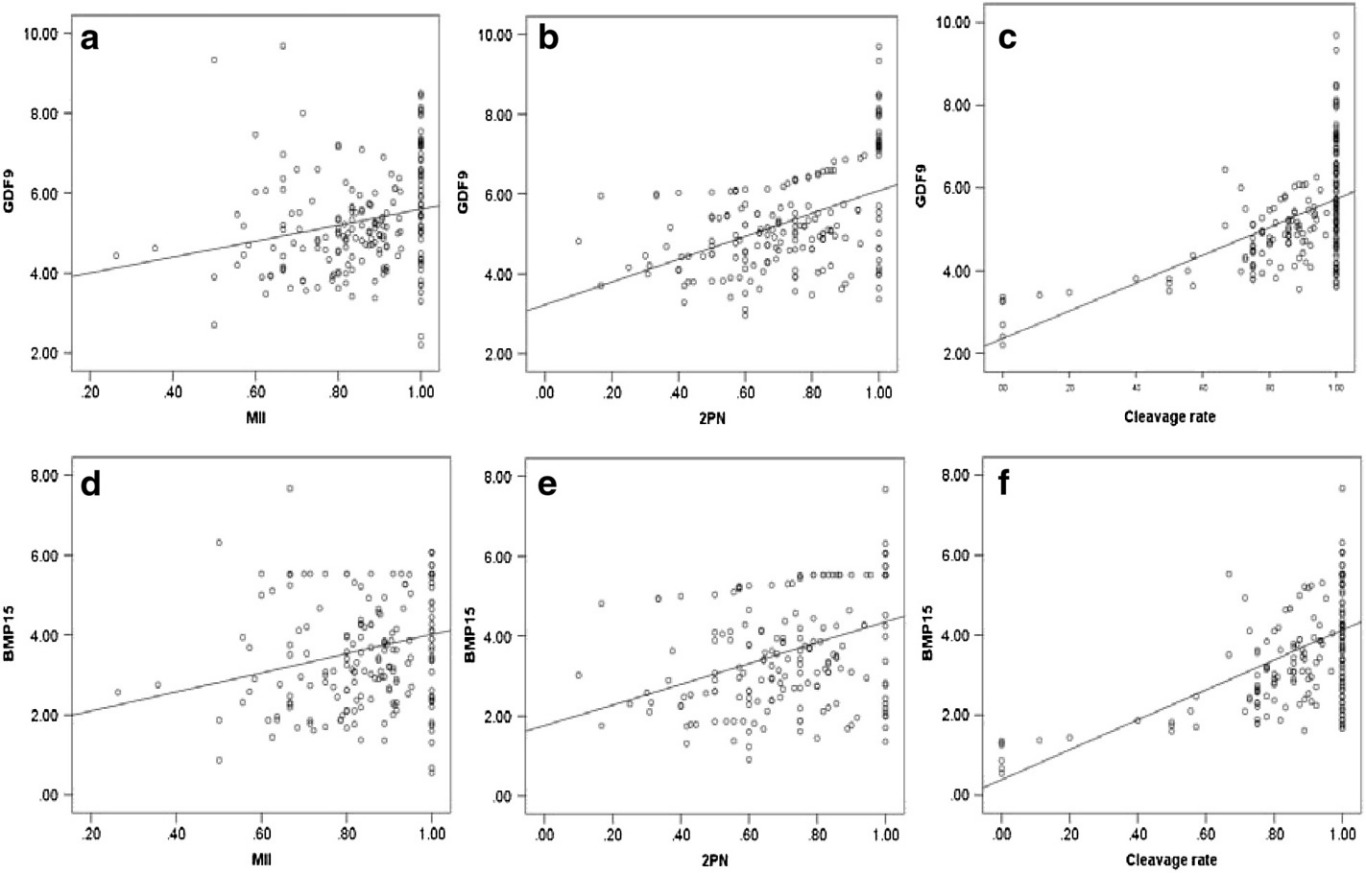
**Partial correlation anlysis was used to analyze the correlation between** ***GDF9*** **and** ***BMP15*** **expression and oocyte developmental potential in 196 patients who underwent ICSI.** The numbers on the horizontal axes refer to the oocyte maturation rate (MII), normal fertilization rate (2PN), and cleavage rate. The numbers on the longitudinal axes refer to the relative mRNA expression of *GDF9* or *BMP15*. **(a)** The correlation between oocyte maturation rate and the relative expression of *GDF9* mRNA. **(b)** The correlation between normal fertilization rate and the relative expression of *GDF9* mRNA. **(c)** The correlation between cleavage rate and the relative expression of *GDF9* mRNA. **(d)** The correlation between oocyte maturation rate and the relative expression of *BMP15* mRNA. **(e)** The correlation between normal fertilization rate and the relative expression of *BMP15* mRNA. **(f)** The correlation between cleavage rate and the relative expression of *BMP15* mRNA.

The expression level of *GDF9* mRNA was significantly associated with oocyte maturation, normal fertilization, and cleavage rate after age adjustment; and the partial correlation coefficients were 0.353 (*P* < 0.001), 0.489 (*P* < 0.001), and 0.592 (*P* < 0.001), respectively (Figure 2a–c). Similarly, the expression level of *BMP15* mRNA was also associated with oocyte maturation, normal fertilization, and cleavage rate after age adjustment; and the partial correlation coefficients were 0.345 (*P* < 0.001), 0.402 (*P* < 0.001), and 0.593 (*P* < 0.001), respectively (Figure 2d–f).

### Comparison of the expression levels of *GDF9*and *BMP15*mRNAs between the groups with and without high-quality embryos

**Figure 3:**
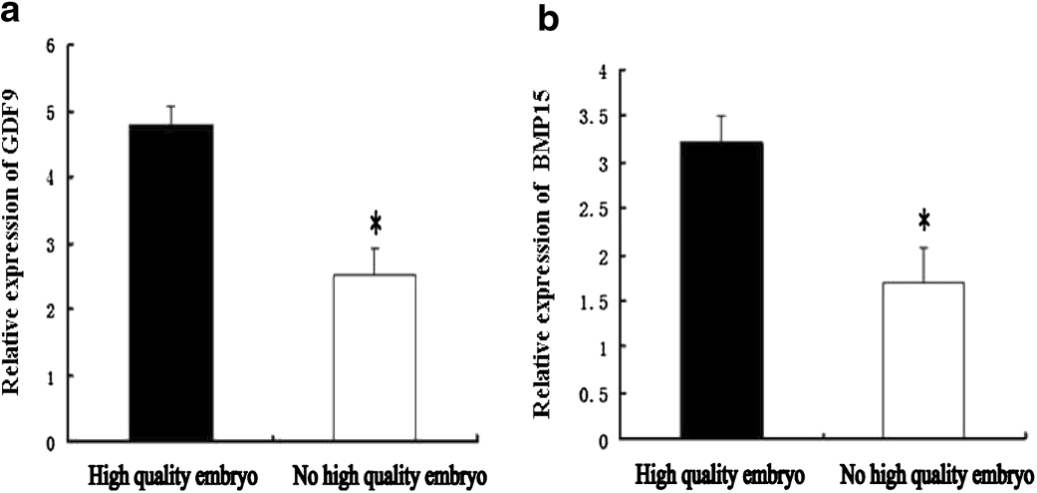
**Comparison of OSFs mRNA levels between the groups with and without high-quality embryo.** The relative expression of *GDF9* mRNA **(a)** in the groups with high-quality embryos. **(b)** The relative expression of *BMP15* mRNA in the groups without high-quality embryos. *P<0.05.

The patient information is presented in Table 2. The relative expression levels of *GDF9* and *BMP15* mRNAs in the group with high-quality embryos were 4.79 ± 0.27 and 3.21 ± 0.30, respectively, while their levels in the group without high-quality embryos were 2.52 ± 0.39 and 1.69 ± 0.39, respectively. Thus, the expression levels of *GDF9* (*P* < 0.05, *F* = 20.62) and *BMP15* mRNAs (*P* < 0.05, *F* = 2.75) in the group with high-quality embryos were signifcantly greater than those in the group without high-quality embryos after adjusting for the number of retrieved oocytes (Figure 3a–b).

**Table 2:** **Clinical parameters of the subjects with and without high-quality embryos**

Variable	High-quality embryos	No high-quality embryos	*P*
*Cases*	106	90	-
*Age (years)*	31.58 ± 4.91	32.29 ± 5.15	ns
*BMI (kg/m* ^*2*^ *)*	21.35 ± 2.63	21.75 ± 3.00	ns
*Antral follicle count (both ovaries)*	15.28 ± 6.25	12.28 ± 6.76	ns
*Ovary volume (mL)*	5.92 ± 2.46	5.57 ± 3.44	ns
*FSH (IU/L)*	8.24 ± 2.45	9.56 ± 5.72	ns
*LH (IU/L)*	5.30 ± 3.18	5.11 ± 3.34	ns
*E*_*2*_ *(pg/mL)*	50.81 ± 24.95	62.48 ± 26.68	ns
*T (ng/L)*	1.34 ± 0.60	2.19 ± 0.76	ns
*PRL (pg/mL)*	17.27 ± 11.90	15.82 ± 7.07	ns
*Sperm number (×10* ^*6*^ */ml)*	0.52 ± 0.12	0.49 ± 0.15	ns
*Sperm motility (%)*	2.82 ± 0.83	3.56 ± 1.82	ns
*Days of stimulation*	11.20 ± 2.87	10.42 ± 3.94	ns
*Gonadotropin dose (IU)*	2149.38 ± 893.95	2167.46 ± 1112.80	ns
Retrieved oocytes*	14.39 ± 4.49	9.79 ± 3.75	<0.01
*Oocyte maturation rate (%)*	85.06 ± 16.66	82.26 ± 18.47	ns
Normal fertilization rate (%)*	75.09 ± 17.42	65.29 ± 29.80	<0.01
Cleavage rate (%)*	91.67 ± 10.35	81.02 ± 29.21	<0.01
Pregnancy rate (%)*	58.49	42.22	<0.01

*P < 0.05, the group with high-quality embryos compared with the group without high-quality embryos; ns: not significant.

### Comparison of the expression levels of *GDF9*and *BMP15*mRNAs between the groups with or without pregnancy

**Figure 4:**
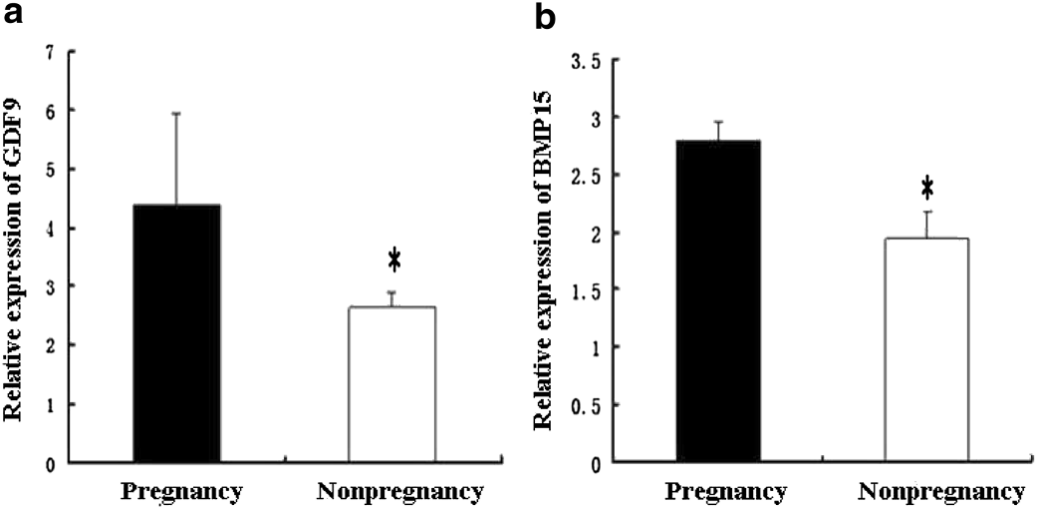
**Comparison of OSFs mRNA levels between the groups with and without pregnancy.** **(a)** The relative expression of *GDF9* mRNA in the pregnancy group and the nonpregnancy group. **(b)** The relative expression of *BMP15* mRNA in the pregnancy group and the nonpregnancy group. *P<0.05.

The patient information is presented in Table 3. The expression level of *GDF9* mRNA in the pregnancy group (4.40 ± 1.55) was significantly greater than that in the nonpregnancy group (2.65 ± 0.24) (*P* < 0.05, *F* = 18.35, Figure 4a). Also, the *BMP15* mRNA level in the pregnancy group (2.79 ± 0.17) was significantly greater than that in the nonpregnancy group (1.93 ± 0.25) after adjusting for the number of retrieved oocytes (*P* < 0.05, *F* = 1.96, Figure 4b).

**Table 3:** **Clinical parameters of the subjects with and without pregnancy**

Variable	Pregnancy	Nonpregnancy	*P*
*Cases*	100	96	-
*Age (years)*	30.74 ± 4.42	31.08 ± 5.16	ns
*BMI (kg/m* ^*2*^ *)*	21.19 ± 3.60	21.68 ± 2.76	ns
*Antral follicle count (both ovaries)*	15.63 ± 6.23	11.94 ± 6.74	ns
*Ovary volume (mL)*	6.12 ± 3.18	5.34 ± 2.74	ns
*FSH (IU/L)*	8.30 ± 2.41	9.38 ± 5.63	ns
*LH (IU/L)*	5.19 ± 3.16	5.19 ± 3.39	ns
*E*_*2*_ (pg/mL) *	48.33 ± 37.49	64.46 ± 37.95	<0.01
*T (ng/L)*	1.73 ± 0.92	1.74 ± 0.79	ns
*PRL (pg/mL)*	15.91 ± 7.71	17.36 ± 8.97	ns
*Sperm number (×10* ^*6*^ */ml)*	0.35 ± 0.16	0.42 ± 0.18	ns
*Sperm motility (%)*	2.65 ± 0.63	2.85 ± 0.79	ns
Days of stimulation*	11.41 ± 2.33	10.24 ± 4.19	0.016
*Gonadotropin dose (IU)*	2212.09 ± 848.13	2089.00 ± 1127.21	ns
Retrieved oocytes*	14.14 ± 4.13	10.38 ± 3.31	<0.01
*Oocyte maturation rate (%)*	84.30 ± 13.67	83.38 ± 20.78	ns
*Normal fertilization rate (%)*	71.47 ± 17.93	69.63 ± 29.89	ns
Cleavage rate (%)*	90.05 ± 13.30	83.04 ± 27.96	0.024

*p < 0.05, the pregnancy group compared with the nonpregnancy group; ns: not significant.

### Diagnostic value of GDF9 and BMP15 mRNAs for predicting pregnancy

The diagnostic value of GDF9 and BMP15 mRNAs for predicting pregnancy is summarized in Table 4. The area under the ROC curve of GDF9 mRNA for predicting pregnancy was 0.816 (0.757–0.875), with a cut-off value of 4.82, a sensitivity of 82%, and a specificity of 64%. The area under the ROC curve of BMP15 for predicting pregnancy was 0.746 (0.671–0.821), with a cut-off value of 2.60, a sensitivity of 78%, and a specificity of 52%.

**Table 4:** **Diagnostic values of** ***GDF9*** **and** ***BMP15*** **for predicting pregnancy**

OSFs	AUC	*P*value	Threshold	Sensitivity	Specificity
*GDF9*	0.816 (0.757–0.875)	<0.001	4.87	82%	64%
*BMP15*	0.746 (0.671–0.821)	<0.001	2.60	78%	52%

## Discussion

In the present study, a qPCR method was used to detect the expression levels of *GDF9* and *BMP15* mRNAs in cumulus granulosa cells from patients who underwent ICSI. The correlation between *GDF9* or *BMP15* mRNA and oocyte development potential was analyzed in order to explore new biomarkers for embryo selection.

OSFs are not only autocrine but also paracrine factors. GDF9 and BMP15 were examined to predict oocyte developmental potential, while previous studies have only detected the expression levels of some downstream genes [22, 23]. Many studies have confirmed that both GDF9 and BMP15 are expressed in the oocyte cytoplasm and cumulus granulosa cells [19–21]. In animal studies, it has been shown that GDF9 and BMP15 can stimulate oocyte development [24]. Furthermore, mouse oocytes matured with exogenous GDF9 had a higher percentage of hatching blastocysts and a better blastocyst quality, and the number of viable fetuses was also increased [25]. GDF9 and BMP15 play an important role in oocyte development. Both factors contribute to promoting the proliferation and metabolism of granulosa cells, and stimulate the expression of kit ligand (KL) on granulosa cells. The KL acts on its receptor on the oocyte and modulates oocyte development [26]. To date, the expression of *GDF9* and *BMP15* mRNAs has not been used to predict oocyte quality. Here, we found that the expression of *GDF9* and *BMP15* mRNAs in cumulus granulosa cells was positively correlated with oocyte maturation, normal fertilization rate, and cleavage rate. Because cumulus granulosa cells are closely associated with the oocyte, the expression of *GDF9* and *BMP15* mRNA in cumulus granulosa cells may reflect oocyte developmental potential.

In addition, we observed that the mRNA expression of *GDF9* and *BMP1*5 were positively associated with age and BMI. Age and BMI are important factors that may affect the ovarian response and pregancy outcome of assisted reproductive technology. However, the age and BMI may not have a direct relationship with the expression of *GDF9* and *BMP15*; therefore, further investigation is needed. Furthermore, the rate of oocyte maturation in the group with higher expression of *GDF9* and *BMP15* mRNA was significantly greater than that in the group with lower *GDF9* and *BMP15* mRNA expression. The rate of oocyte maturation was positively related to the mRNA expression of *GDF9* and *BMP15*. Fertilization is closely related to oocyte quality such as oocyte maturation and spindle structure [27–30]. The fertilization rate in high-quality oocytes is often greater than that in low-quality oocytes. Low-quality oocytes tend to have a higher abnormal fertilization rate due to imperfect oocyte function. Our results indicated that the fertilization rate increased along with the increased mRNA levels of GDF9 and BMP15. Moreover, oocyte quality dramatically affects subsequent embryo development. Low-quality oocytes often have a lower chance of developing into high-quality embryos [31]. Accordingly, the patients with a higher expression of OSFs also had a higher cleavage rate. This evidence helps to explain our findings that the expression of *GDF9* and *BMP15* mRNAs in the group with high quality embryos was significantly greater than that in the group without a high quality embryo. Clinical pregnancy is an important indicator for evaluating embryo quality. Although the pregnancy outcome is determined by several factors such as sperm quality and the uterine endometrium, the oocyte is the most important factor for clinical outcome. A high-quality oocyte more easily develops into a high-quality embryo; accordingly, the chance of clinical pregnancy is also increased [32]. The present study confirmed that the expression of *GDF9* and *BMP15* mRNAs in the pregnancy group was significantly greater than that in the nonpregnancy group, suggesting that the expression of *GDF9* and *BMP15* mRNA may be used as an indicator to predict clinical pregnancy outcome. As the ROC curve was used to evaluate the diagnostic value of *GDF9* and *BMP15* mRNAs, the cut-off value can be established for predicting pregnancy with a relatively high sensitivity. These data indicate that the detection of *GDF9* and *BMP15* mRNAs in cumulus granulosa cells may have broad application prospects as invasive biomarkers for evaluating ooctye developmental potential.

However, the limitation of this study is that the cumulus granulosa cells were not harvested from an individual oocyte, so the expression of *GDF9* and *BMP15* mRNA does not match with each oocyte and embryo. A future study that detects the expression of OSFs from an individual follicle is still needed to validate the conclusion. Additionally, there may be some other OSFs such as BMP6 and fibroblast growth factor (FGF) 8B that could be used as biomarkers [33, 34]. In the present study, we only detected the mRNA expression of the two well-known factors *GDF9* and *BMP15* in cumulus granulosa cells. It will be interesting to explore the relationship between the mRNA expression of other OSFs and oocyte developmental potential in the future.

## Conclusions

The expression of *GDF9* and *BMP15* mRNAs was significantly correlated with oocyte maturation, fertilization, embryo quality, and clinical pregnancy outcome. Thus, the levels of *GDF9* and *BMP15* mRNA in cumulus granulosa cells may be considered as new biomarkers for predicting oocyte developmental potential.

## Abbreviations

E_2_: Estradiol
FSH: Follicle-stimulating hormone
LH: Luteinizing hormone
PRL: Prolactin
T: Testosterone.
